# *Ternidens deminutus* Revisited: A Review of Human Infections with the False Hookworm

**DOI:** 10.3390/tropicalmed4030106

**Published:** 2019-07-18

**Authors:** Richard S. Bradbury

**Affiliations:** Slovak Tropical Institute, St. Elizabeth University, 81101 Bratislava, Slovakia; rbradbur76@gmail.com

**Keywords:** *Ternidens*, ternidensiasis, false hookworm, hookworm, soil transmitted helminths, STH, helminth, zoonosis, human, primate

## Abstract

*Ternidens deminutus*, the false hookworm of humans and non-human primates, represents a truly neglected intestinal helminth infection. The similarity of the eggs of this nematode to those of hookworm both presents a diagnostic challenge and a potential confounder in prevalence surveys of soil transmitted helminths (STH) in regions where *T. deminutus* is found. The helminth infects non-human primates throughout Africa and Asia, but reports of human infection are almost exclusively found in eastern and southern Africa. Historically, an infection prevalence up to 87% has been reported from some parts of Zimbabwe. Scarce reports of ternidensiasis have also been made in individuals in Suriname and one from Thailand. Little work has been performed on this parasite since the 1970s and it not known why human infection has not been reported more widely or what the current prevalence in humans from historically endemic areas is. This review serves to revisit this enigmatic parasite and provide detail to a modern audience of parasitologists on its history, clinical presentation, geographic distribution, life cycle, biology, morphology, diagnosis and treatment.

## 1. Introduction

The World Health Organization’s (WHO) global target to eliminate morbidity due to soil-transmitted helminths in children by 2020 [1] has resulted in increased interest within the global health community towards the control of STH (the hookworms, *Ascaris lumbricoides* and *Trichuris trichiura*). This has included increased activity in the global surveillance of hookworm and other STH prevalence [2]. Surveillance activities in most countries still rely on microscopic detection and identification of eggs. Discrepancies between microscopy and PCR results in some of these surveys have highlighted the existence of human infecting helminths having eggs that may be morphologically confused with those of STH [3]. This has raised the need to revisit the intestinal helminths of humans that are historically known to occur at moderate to high prevalence in some populations and are having eggs morphologically similar to those of other STH.

*Ternidens deminutus* (the false hookworm) is one such helminth, with infected humans passing eggs that are easily confused with hookworm eggs. Surveys of human intestinal helminths in Zimbabwe during the 1970s revealed a prevalence of this parasite of up to 87% in some populations [4]. The remarkable similarity of *T. deminutus* eggs to those of hookworms often confounded previous surveys of hookworm prevalence in these regions [5]. Furthermore, *T. deminutus* infection has not been reported from humans in any surveillance studies for the past 25 years [6], nor in any individual case report since 2005 [7]. In areas where prevalence of this worm was historically high, such as southern Zimbabwe, this is likely due to misidentification of *T. deminutus* eggs as those of hookworms, rather than elimination of the parasite from these communities. It appears to be time to revisit *Ternidens* and to inform the current generation of parasitologists and epidemiologists of the existence, diagnosis and treatment of this neglected tropical disease.

Several countries, regions and cities referred to in this review have changed their names multiple times over the past one hundred years. To avoid confusion for readers, the place names current at the time of writing have been used throughout.

## 2. History

*T. deminutus* (nematoda: Strongylidae) is an intestinal helminth of primates, including humans, monkeys, gorillas and baboons in Africa and Asia (Table 1). The species was first described by Railliet and Henry (1905) [8] when reviewing two museum specimens taken at autopsy in 1865 by the French naval physician Moniester from the intestine of a patient native to Mozambique (but living on the island of Mayotte) who had died of anemia. Originally incorrectly identified as *Ancylostoma duodenale* by Moniester, Railliet and Henry differentiated the worms on the basis of morphology and described them as *Triodontophorus deminutus* [8]. In 1908, Turner found upon autopsy a number of female worms in the large intestine of two patients from Malawi who had died working in the mines of Johannesburg (South Africa). These worms were distinct from the *Necator americanus* specimens found in the same patient’s small intestine, both in their morphology and that the site of infection was the large intestine. Leiper (1908) [9] examined these worms and identified them as *T. deminutus* [10]. The following year, Railliet and Henry (1909) [11] revised the taxonomy of the family *Strongylidae* and the name *Triodontophorus* was suppressed as a synonym for a new genus, *Ternidens*. Stannus sent a number of worms recovered post-mortem from the intestines of prisoners from Loma, Malawi to Leiper who identified them as *T. deminutus*, though these particular cases were not reported in the literature until they were communicated by Sandground in 1931 [12]. 

Infection in primates was first reported by Leiper, in a western lowland gorilla (*Gorilla gorilla gorilla*) taken from Gabon which died at the London zoological gardens. Between 1906 and 1937, infection was identified in numerous species of monkeys from Africa and Asia, as well as in a baboon (*Papio ursinus griseipes*) and a chimpanzee (*Pan troglodytes versus*) [13]. Smith, Fox and White (1908) [14] described a new worm, *Globocephalus macaci* in a pig-tailed monkey (*Macaca nemestrina nemestrina*) which died at the Philadelphia zoo, but this worm was considered to be *T. deminutus* by Sandground [12].

The significance of *T. deminutus* as a human pathogen became more widely recognized following the work of Sandground in Zimbabwe in the late 1920s [12,15]. Sandground first identified a novel and unusual helminth egg in the feces of an American medical missionary working in the Mount Selinda region of that country. He first considered this nematode as possibly belonging to the genera *Trichostongylus* or *Oesophagostomum*. However, on receipt of several fecal samples and adult worms taken from people in the immediate vicinity of the mission where the patient worked, the parasites were identified as *T. deminutus* [15]. 

In response to this finding, Sandground traveled to Southern Africa and carried out surveys for *T. deminutus* infection on workers at the City Deep Goldmine in Johannesburg, the Mount Selinda and Chikore regions of south east Zimbabwe, Livingstone in Zambia and the Gogoyo region and Maputo city regions of Mozambique. Based on results from the NaCl passive flotation technique, *Ternidens* infection prevalence of over 50% was reported from some regions, while in others few or no *T. deminutus* infections were identified. In Johannesburg, 15 of 503 individuals (3%) were found to be infected with *T. deminutus*, eleven of whom were from Mozambique, although some patients from the Eastern Cape and Gauteng region of South Africa were also found to be infected [15]. At Mt. Selinda, 112/190 (59%) of individuals tested harbored *T. deminutus*, either alone (n = 48) or in co-infection with hookworms (n = 64). At Chikore, 8/12 (67%) were infected, while at Gogoyo, 34/124 (27%) were infected, with all but one being co-infected with hookworms. No *T. deminutus* infections were found amongst 54 individuals in Livingstone. Of 323 individuals from many parts of Mozambique examined in Maputo, only one was infected with *Ternidens* [12]. At Mount Selinda, infection intensity of between 21 and one hundred individual worms was identified in some patients following treatment with carbon tetrachloride, terachloroethylene or a combination of these drugs with oil of chemopodium [12].

Following Sandground’s work, Blackie reported that prevalence of *T. deminutus* infection was between 5.3% and 16% in various parts of Zimbabwe [16], while Van den Berghe (1934) [17] reported a prevalence of 15% in a survey of people in the Katanga region of the Democratic Republic of Congo. A single case was reported from Zambia by Blackie in 1932 [16] and from Mauritius in 1937 by Webb [18]. Forty-four adult worms were provided to the US Naval Medical School in 1947 by the Central Medical Laboratories in Maputo, Mozambique [13]. Several hookworm surveys in Zimbabwe by Gelfand between 1945 and 1965 failed to detect the parasite, which was later suggested as being possibly due to failure to differentiate the eggs from those of hookworm rather than due to its absence in the populations sampled [5].

## 3. Biology and Life Cycle

The closest relative to of the genus *Ternidens* is *Oesophagostomum* [19,20], another nodular intestinal worm affecting both humans and non-human primates in Africa and Asia. Only two species are currently recognized in the genus; *T. deminutus* and *Ternidens simiae*. *T. deminutus* has been reported in monkeys, baboons and humans in Africa and Asia (Table 1). The average size of *T. deminutus* adults in humans is larger than that of baboons [21]. *T. simiae* has been reported once from the gut of a monkey in Sulawesi, Indonesia [22], but could not be confirmed [23]. Genetic variations between *T. deminutus* from different monkey hosts has raised the possibility that several cryptic species may infect different host primates [19,20].

Infection of the definitive host may occur via oral ingestion of third stage (L3 filariform) larvae [24], which establish parasitism in the large intestine, particularly the colon, but also the cecum, compared to hookworms, which are primarily parasites of the duodenum. These L3 larvae are thought to then enter the intestinal mucosa, form nodules in which they mature to L4 larvae and then re-enter the lumen as adults to mate [12,24]. Based on observations of blood in the intestine of adult worms [21] combined with histochemical and biochemical analysis of the contents of worm guts [25] that adult worms possibly ingest blood. Whether they are true blood suckers or simply ingest blood oozing from lesions that they have created in the intestinal mucosa remains unclear [21]. 

Infections in human subjects examined by Goldsmid [26] found a mean worm load of 22.7 ± 5.9 worms per subject and a mean egg output of 494.4 ± 158.4 eggs per gram of feces, with a mean egg production of approximately 3500 and 7000 eggs/worm per day [26]. Eggs are passed with eight stage (rarely four stage) morulae in the feces [21]. The female:male ratio in both baboons and humans was 1:6 [26]. Eggs become fully mature 24 to 30 h after passage and the L1 (rhabditiform larvae) hatch after 48-72 h. The L1 rhabditiform larvae develop into L2 stage after two to three days at 29 °C. Development to L3 filariform larvae at this temperature takes eight to ten days [26]. The L3 larvae of *T. deminutus* appear to be relatively environmentally resistant. Sandground claimed to have revived larvae following three days of desiccation [12]. Experiments by Goldsmid found that approximately 4% of L3 larvae survived and recovered motility after 24 h of desiccation at 14 °C at 60% relative humidity. After 24 h in these desiccated conditions, no larvae survived [26]. The morphology of revived larvae following desiccation was greatly affected, with many losing their sheath, developing vacuoles in the cuticle and internal structures becoming unrecognizable [12,26], and thus the infectivity of such affected larvae is not reliable. Resistance to cold was also observed, with over 60% of 224 larvae surviving at 5 °C for ten days. Survival continued for 70 days, though the percentage of viable larvae rapidly declined by this time. No viable larvae were detected at 84 days. Larvae did not survive freezing at –5 °C for one hour [26]. As is seen with hookworm infection, the passage of *T. deminutus* eggs in feces shows a seasonal prevalence, with the highest rates of detection in Zimbabwe being during the Summer months, which are characterized by high temperatures and rainfall [20]. When 301 patients between the ages of 0–2 and >65 years of age were examined, egg output was greatest in patients between the ages of 7 and 35 years [20].

Although a direct life cycle is assumed, the failure of attempts to infect volunteers through ingestion of filariform larvae or by transdermal inoculation [12,15,16] also led to the proposal that an insect intermediate host may be involved in transmission [13,21,27]. Termites were suggested as such a possible host due to their being shared in the diet of both humans and monkeys in areas where human infection is common [28]. 

*T. deminutus* is thought to be a zoonosis acquired from non-human primates, although some “spillback” from humans to monkey populations may also occur in some areas [21]. Attempts to infect humans via oral ingestion of larvae cultured from a baboon by Sandground [12] were unsuccessful and some have suggested that humans may be the main host species [12,29]. The possible presence of multiple cryptic species might explain this controversy, with a human specific haplotype of the parasite existing alongside several host-specific non-human primate genotypes. This theory is supported by the analogous presence of several host-specific haplotypes, including a human-specific haplotype, in the genetically similar helminth species *Oesophagostomum bifurcum* [30]. The average size difference between adults in humans and baboons might support this, though such morphological variation in size between hosts is not uncommon in nematode species. This hypothesis would also explain the almost complete absence of human infections in Asia, despite the parasite being prevalent in non-human primates in the continent. 

## 4. Geographic Distribution and Prevalence

**Figure 1:**
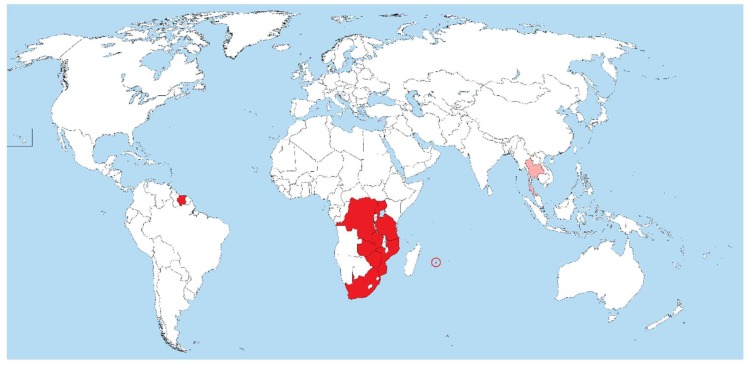
Map of the world showing countries (in red fill) where *Ternidens deminutus* infection in humans has been confirmed (red circle = Mauritius; pink fill = single case report, possible misidentification of *Oesophagostomum* infection).

*Ternidens* infections of monkeys, chimpanzees and baboons have been reported throughout sub-Saharan Africa and Asia (Table 1). Infections have thus far not been reported in new world primates. In humans, reports of infection have been almost exclusively from sub-Saharan Africa, with only one case report from Thailand and two from Suriname (Figure 1). 

Human *T. deminutus* infections often occur in high prevalence foci, such as seen in several villages of Zimbabwe. In 1972, Goldsmid, undertook to revisit the work of Sandground [12] on *T. deminutus* and actively surveyed patients at the Harari Central Hospital in Harare, Zimbabwe. Of 5545 patients examined, 208 (3.75%) harbored infections [5]. Several further surveys in eastern and the central north of Zimbabwe (Bindura, Chiweshe, Burma Valley, Masvingo, Nyanga, Lundi, Maramba, Mount Selinda, Sabi Valley, Harare, Triangle and Mutare) by Goldsmid found a wide range of prevalence, between 0% and 87% (mean average 19%; median 9.2%), in humans. Of 32 baboons tested in Bikita and Marimba, over 70% were infected. No human cases were found in Kariba, in the North East Zambezi Valley area of the country [31]. A later survey in the Kadoma region of central Zimbabwe by Goldsmid found only 0.7% of 595 people infected [32]. In these surveys, Goldsmid applied the same NaCl passive flotation technique as Sandground had used in 1931 [12], with *T. deminutus* identification based on egg size and any difficult to differentiate eggs cultured by the Harada-Mori technique to allow definitive identification of the L3 larvae [5,31,32].

A survey of 4500 people from the Songea district of Tanzania found *T. deminutus* (identification based on egg size only, technique not stated) in 105 subjects [33]. In a report of 34 cases of intestinal helminthoma from Entebbe, Uganda in 1972, one case of *T. deminutus* helminthoma of the ileum was confirmed [34]. Two *Ternidens* infections, identified based on the morphology of the adult worms passed after treatment, in school children from Zimbabwe were reported in a study of the efficacy of albendazole as a hookworm treatment by Bradley in 1990 [35]. A further survey of people in the Burma Valley region of Zimbabwe in 1993 using quadruplicate Kato Katz examination, and identifying *T. deminutus* based on egg size alone, identified 5% as having *T. deminutus* infection [36]. 

Only one human *T. deminutus* case has been reported from Asia. This infection occurred in Thailand in 1983, but was not reported until 2002. A helminth was identified upon histology of an intestinal nodule taken from the colon of a 33 year old Thai female as an immature adult male *T. deminutus* [7]. In this case, the species identification and differentiation from the clinically and morphologically similar *Oesophagostomum* species was determined only by the width of the parasite in the histological section (300–500 µm; max 550 µm). This larger diameter was the only differentiating feature of this stage of *Ternidens* from *Oesophagostomum* in cross section and was the only feature used to make the species identification [7]. However, *Oesophagostomum* species in section may be up to 700 in diameter [37]. Furthermore, the diameter reported was below that reported for another *T. deminutus* (650 µm) infection in section, admittedly only measured in a single adult female [38]. Without other supporting molecular data, the only human case of human ternidensiasis reported from Asia may represent a misidentification of *Oesophagostomum* infection. 

Jozefsoon [6] reported two *T. deminutus* infections among 431 people belonging to a community of descendants of slaves brought from West Africa and living a traditional lifestyle in the southern interior of Suriname (South America). Identification was based on the morphologic identification of larvae in Harada-Mori culture, with the morphologic approaches described being reliable and the report therefore likely to be accurate. This report was particularly unusual as *T. deminutus* has not been reported from non-human primates on that continent. The people with *T. deminutus* infection in Suriname may be the descendants of people that moved from West Africa, with the remnant worm population still circulating amongst this group [6]. Although it remains occasionally reported in non-human primates [39], no more cases of human *Ternidens* infection have been reported in the scientific literature since the two Suriname cases in 1994 [6]. Human *Ternidens* infections have almost certainly not disappeared since this time, rather, it seems likely that when encountered, they are being misidentified as hookworm or *Oesophagostomum*.

## 5. Morphology

**Figure 2:**
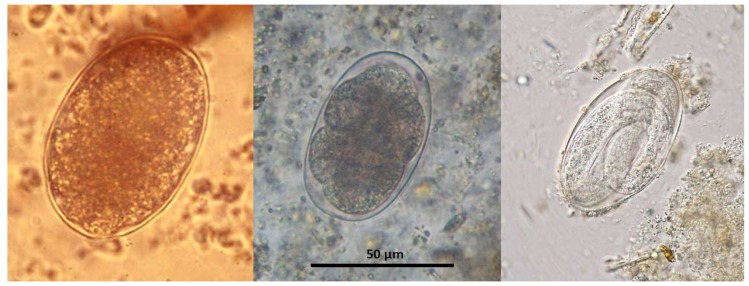
Eggs of *T. deminutus* (left – photographs courtesy of Emeritus Professor John Goldsmid), *Necator americanus* (middle - photograph by Richard Bradbury) and *Oesophagostomum* sp. (with larva developing within – photograph courtesy of CDC DPDx – https://www.cdc.gov/dpdx).

**Figure 3:**
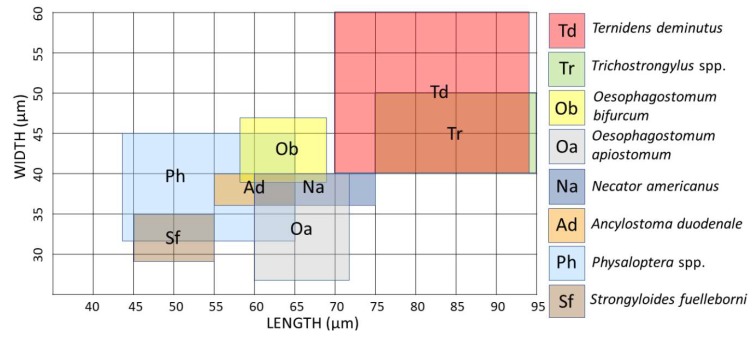
Size range comparison chart of hookworm-like eggs passed in human feces [21,40,41,42].

The thin shelled eggs of *T. deminutus* superficially resemble those of hookworms (Figure 2). Eggs are also distinguished from those of hookworms by their larger size (70–94 µm × 40–60 µm) [21] and greater ratio of width to length (Figure 3) [12]. Eggs may have between four and 32 morulae, which will further develop into larvae within the egg and hatch. 

**Figure 4:**
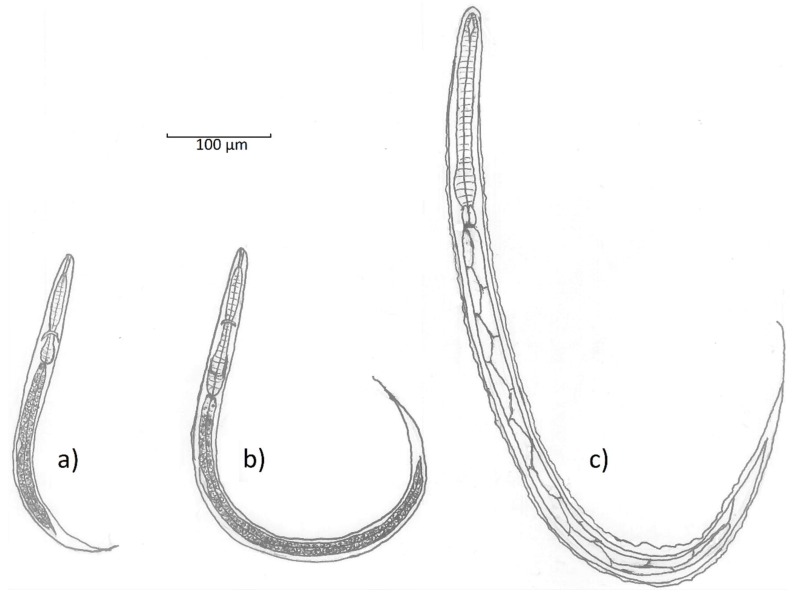
Line drawings showing the morphology of **a**) rhabditiform (L1) larva; **b**) rhabditiform (L2) larva and **c**) filariform (L3) larva of *Ternidens deminutus* (line drawings by Richard Bradbury).

The first stage (L1) rhabditiform larvae of *T. deminutus* measure between 300 and 360 µm long by ~20 µm at the widest point [12] (Figure 4a). The cylindrical buccal cavity is 10.5 × 1.5 µm in length and breadth and the esophagus approximately 95 µm long. A refractile, spindle shaped genital primordium is 11.2 µm long. At the distal end, a long flagella-like tail reaches 70 µm in length [12]. L2 larvae are longer (620 µm) and wider (32 µm) with an esophagus 140 µm in length (Figure 4b). While these rhabditiform larvae appear similar to those of the hookworms and *Strongyloides stercoralis*, they may be differentiated by observing the combination of the long buccal cavity, prominent genital primordium, and longer tail. 

The filariform (L3) stage larvae of *T. deminutus* (Figure 4c) are easily distinguished from other “hookworm-like” larvae derived from humans. This life stage measures between 630–730 µm long by 29–35 µm wide and is most distinguished by the palisade “zig-zag” appearance of the gut, shared with larvae of *Oesophagostomum*. The larval cuticle is distinctly striated and six minute, punctiform, papillae may be found on the head [12]. The head has an indentation at the entrance to the spear shaped remnant buccal cavity [21]. The esophagus measures 150-165 µm in length and is almost uniform in length and width, but for a slight bulge distally. The esophagus to intestine ration is approximately 1:3 [26]. Two elongate sphincter cells divide the esophagus from the intestine and the intestine is composed of at least ten pairs of large triangular cells which provide the palisade appearance of this organ. A genital primordium 15 µm long is found hugging the intestinal wall near the middle of the larva. The anus opens between 120-145 µm from the end of the tail [12]. The tail tapers to a fine point and the filamentous end of the sheath extends some distance further, appearing threadlike at the posterior extremity [6,12]. This morphologic appearance most closely resembles *Oesophagostomum* species L3 larvae, but the two may be differentiated by the greater overall length (702–950 µm), the “Y” shape of the remnant buccal cavity, the far wider and more prominent, rhabditoid esophageal bulb and the absence of sphincter cells between the esophagus and the intestine in *T. deminutus* L3 larvae. There is a much shorter distance from anus to the tip of the tail (45-88 µm) and the rounded tail of *Oesophagostomum* [6,40]. Jozefsoon [6] noted if the distance from the tip of the tail to the tip of the sheath is greater than the distance from the anus to the tip of tail, a larva is likely to represent an *Oesophagostomum* sp. and not *T. deminutus*.

**Figure 5:**
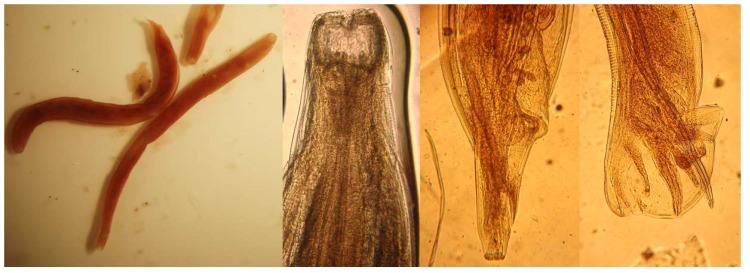
(left to right) adult *Ternidens deminutus* whole female and male worms from a human; anterior of an adult showing the buccal capsule, posterior of an adult female; posterior of an adult male showing copulatory bursa (photographs by Richard Bradbury).

Adult female *T. deminutus* from baboons are 5–12 mm in length (mean 8.60 ± 0.19 mm). Specimens from human hosts measure 9–17 mm in length (mean 11.56 ± 0.81 mm). Adult males are between 4.5–11 mm in length (mean 7.96 ± 0.21 mm) from baboons and 6–13 mm in length (mean 9.67 ± 0.66 mm) from human hosts. Specimens from humans appear to be darker in color than those from baboons [23]. Adult *Ternidens* are straight, unlike the curved appearance of adult hookworms. The cuticle is opaque and a transverse cuticular fold may be seen immediately distal to the buccal capsule. The entire cuticular surface has transverse striations. The large, swollen sub-globose buccal capsule contains an anteriorly facing mouth surrounded by a collar and 22–24 bristles of the corona radia (Figure 5). Four knob shaped sub median papillae and two lateral amphids are present on the anterior surface of the worm. On the base of the buccal capsule are three deep set teeth with two lateral and one central lamellae. The esophagus measures 525–840 (mean 739) µm in human derived worms and 511–837 (mean 727) µm in baboon derived worms. In females, the posterior tapers to a vulva and anus. The distance between the vulva and anus is 372–558 (mean 406) µm in human derived worms and 232–418 (mean 359) µm in baboon derived worms. In male worms, the anterior splays out into a cup shaped bursa consisting of rays encircling two copulatory spicules (Figure 5). A gubernaculum is present. The spicules measure 1116–1441 (mean 1267) µm in human derived worms and 1023–1302 (mean 1178) µm in baboon derived worms [23]. One description of the recovery of a rare deformed adult male *Ternidens* with bifurcated anterior was made by Lyons and Goldsmid in 1973 [43].

## 6. Clinical Presentation

Ternidensiasis most commonly presents in a similar manner to oesophagostomiasis [19], with multiple intestinal abscesses, nodules or helminthomas of the large intestine [21]. Adult worms may also be found free in the intestinal lumen attached to the intestinal mucosa [26]. The clinical effects of infection have not been well studied. Infection appears to be asymptomatic in many cases. In some infections with heavy worm loads associated malaise, obstipation [13] and microcytic hypochromic anemia [21] have been reported. Due to the co-infections with other helminths and the poor nutritional condition of the participants involved in many studies, it is difficult to accurately discriminate the contribution of *Ternidens* infection to anemia as opposed to the effects of other chronic diseases and other parasitic infections. 

## 7. Laboratory Diagnosis

The diagnosis of *Ternidens* has most commonly been undertaken by measurement of size and differentiated from hookworm based on the size of the eggs [21]. Eggs may be found in direct preparations, but recovery is increased by the use of techniques such as saturated salt flotation [12,24], Kato Katz [34] and formalin-ethyl acetate sedimentation. Culture of larvae to L3 stage is recommended to allow definitive species identification [6]. Larval culture techniques such as Harada-Mori have been successfully employed to detect and identify *Ternidens* infections [24]. The Koga agar larval culture technique has not yet been applied the detection of *T. deminutus* but given the previous success of Harada-Mori technique, it appears likely that this method would also be successful. Recovery and identification of the adult worms on purgation or autopsy is the gold standard for diagnosis. No diagnostic PCR for the detection of *T. deminutus* has been developed thus far.

## 8. Treatment

As much of the work on *T. deminutus* was performed prior to the advent of benzimidazole derivative and macrocyclic lactone medications, there have been no controlled studies of the efficacy of modern anthelmintics commonly used in mass drug administration campaigns on *T. deminutus* infection. Historically, oil of chemopodium, carbon tetrachloride or tetrachloethylene were administered, but were ineffective [12,31]. Treatment with phenylene diisothyacyanate resulted in only a 22% cure rate and bephenium hydroxynapthoate an 87.5% cure rate [19]. Of the modern drugs available, Thiabendazole yielded a 90.5% cure rate, while Pyrantel pamoate cured 92% of cases [27]. One report has described the efficacy of albendazole when administered to two infected school children in Zimbabwe [34]. This suggests that albendazole may be a suitable treatment for *T. deminutus* infection, but more thorough trials involving albendazole, mebendazole and ivermectin with monitoring for a longer period following treatment would be advisable to comprehensively determine the most effective treatment for infections. In cases where *Ternidens* is causing a bowel helminthoma, surgical intervention may be indicated [34].

## 9. Conclusions

*T. deminutus*, the “false hookworm” has long been an enigmatic and often ignored intestinal helminth of humans. Studies into the worm and the disease caused by it have largely been undertaken by two enthusiastic and capable individuals, specifically John Sandground and John Goldsmid, while little attention was paid to this disease by other researchers. Ternidensiasis is a helminth infection of man capable of causing significant pathology in affected patients, including helminthomas and possibly iron deficiency anemia. No doubt the superficial similarity of the eggs to those of hookworm has led to it being misidentified as hookworm in areas of historically high prevalence, where it most likely remains a significant human helminthiasis and may confound the results of future STH prevalence surveys and control efforts. It is hoped that this paper will educate the modern STH and parasite diagnostics community on the existence and importance of *Ternidens* infection and will encourage further investigation by the community of unusual hookworm-like intestinal helminths into the future.

## Figures and Tables

**Table 1:** Reported non-human primate hosts of *Ternidens deminutus* and their geographic range.

Host name	Common name	Region
*Cercocebus atys*	Sooty mangabey	Africa
*Cercopithecus ascanius schmidti*	Red tailed monkey	Africa
*Cercopithecus campbelli*	Campbell’s monkey	Africa
*Cercopithecus cephus*	Moustached guenon	Africa
*Cercopithecus diana*	Diana monkey	Africa
*Cercopithecus mona*	Mona monkey	Africa
*Cercopithecus petaurista*	Lesser spot-nosed monkey	Africa
*Chlorocebus aethiops centralis*	Grey monkey	Africa
*Chlorocebus pygerythrus*	Vervet (green) monkey	Africa
*Gorilla gorilla gorilla*	Western Gorilla	Africa
*Macaca mulatta*	Rhesus monkey	South East Asia and India
*Macaca nemestrina nemestrina*	Pig tailed macaque	South East Asia
*Macaca nigra*	Black macaque	South East Asia
*Macaca radiata*	Bonnet monkey	India
*Macaca fascicularis fascicularis*	Crab eating macaque	South East Asia
*Pan troglodytes versus*	Chimpanzee	Africa
*Papio anubis*	Olive baboon	Africa
*Papio ursinus griseipes*	Chacma baboon	Africa
*Pongo pygmaeus*	Bornean orang utan	South East Asia
